# Head Position and Posturography: A Novel Biomarker to Identify Concussion Sufferers

**DOI:** 10.3390/brainsci10121003

**Published:** 2020-12-17

**Authors:** Frederick Robert Carrick, Guido Pagnacco, Melissa Hunfalvay, Sergio Azzolino, Elena Oggero

**Affiliations:** 1 Department of Neurology, College of Medicine, University of Central Florida, Orlando, FL 32827, USA; 2 Department of Health Professions Education, MGH Institute of Health Professions, Boston, MA 02129, USA; 3 Department of Research, Centre for Mental Health Research in Association with University of Cambridge, Cambridge CB3 9AJ, UK; 4 Department of Neurology, Carrick Institute, Cape Canaveral, FL 32920, USA; guido@vestibtech.com (G.P.); melissa@righteye.com (M.H.); sergio@azzolino.com (S.A.); Elena@vestibtech.com (E.O.); 5 Electrical and Computer Engineering Department, University of Wyoming, Laramie, WY 82071, USA

**Keywords:** concussion, posturography, proprioception, brain, neck, eye, head, vestibular

## Abstract

Balance control systems involve complex systems directing muscle activity to prevent internal and external influences that destabilize posture, especially when body positions change. The computerized dynamic posturography stability score has been established to be the most repeatable posturographic measure using variations of the modified Clinical Test of Sensory Integration in Balance (mCTSIB). However, the mCTSIB is a standard group of tests relying largely on eyes-open and -closed standing positions with the head in a neutral position, associated with probability of missing postural instabilities associated with head positions off the neutral plane. Postural stability scores are compromised with changes in head positions after concussion. The position of the head and neck induced by statically maintained head turns is associated with significantly lower stability scores than the standardized head neutral position of the mCTSIB in Post-Concussion Syndrome (PCS) subjects but not in normal healthy controls. This phenomenon may serve as a diagnostic biomarker to differentiate PCS subjects from normal ones as well as serving as a measurement with which to quantify function or the success or failure of a treatment. Head positions off the neutral plane provide novel biomarkers that identify and differentiate subjects suffering from PCS from healthy normal subjects.

## 1. Introduction

Balance control systems involve complex systems that direct muscle activity to prevent internal and external influences that may destabilize posture, especially when body positions change [1]. Our functional independence depends upon a postural balance, yet our ability to maintain an upright posture is customarily taken for granted [2]. Postural integrity is obtained through the central nervous system’s integration of sensory afferents and coordinated motor activity, which is dependent upon muscle strength and response to environmental perturbations [3]. Postural stability testing after concussion provides quantitatively valid measures of neurological function [4]. However, measurement error in the quantification of balance affects outcome interpretation and the appropriate development of treatment [5]. Even if the measurements are accurate, there is no guarantee that the measurements will accurately quantify balance performance if necessary functional components are omitted or missed in the testing parameters.

Sports-related concussions are associated with aberrant function of the balance system which depends upon the integration of environmental sensory afferents [6]. It is reasonable to suggest that sensory challenges to balance are included in all clinical evaluations of postural stability. A diagnostic dilemma occurs when attempting to include a spectrum of sensory perturbations that might compromise human stability. Sports concussions are commonly associated with balance impairment, making the use of high-quality assessments of balance an integral part of best practices management of concussions [7]. The computerized dynamic posturography stability score has been established to be the most repeatable posturographic measure using variations of the modified Clinical Test of Sensory Integration in Balance (mCTSIB) [8]. However, the mCTSIB is a standard group of tests relying largely on eyes-open and -closed standing positions with the head in a neutral position and has a probability of missing postural instabilities associated with head positions off the neutral plane.

Head movement is compromised after concussion and clinical applications designed to increase human function involving head–eye vestibular motion therapy (HEVM) have increased balance and decreased multi-system symptoms in post-concussion syndrome (PCS) [9]. It is clear that oculomotor dysfunction after sports concussion is related to head–neck movement pathology, which is also associated with mental health concerns, and eyes-closed postural challenges reveal the greatest pathology of stability [10]. Clinical quantification of balance is dependent upon the metrological characteristics of diagnostic equipment recommended in 2013 by the International Standardization Committee for Clinical Stabilometry of the International Society for Posture and Gait Research (ISPGR) [11]. Clinical judgment and appropriate quantification of function after concussion is dependent upon the integrity of the measurement devices and technology utilized in a clinical practice. The frequency content of the center of pressure pathways measured by computerized dynamic posturography (CDP) is based upon the vertical ground reaction force of a subject translated into a spectral analysis of posturographic diagnostic data [12]. Accurate, reliable instrumentation is central to providing quantification of postural changes that might be associated with changes in environmental stimuli during a postural task.

The center of pressure (CoP) measured by CDP is continuously varying, resulting in changes in mass and rotation speed that allow quantification of areas of support and the frequency of movement force [13]. Since concussion subjects demonstrate aberrations of head and neck movement and stability after concussion, it is reasonable to test different head and neck postures by measuring the CoP and stability scores of a subject. The standardized mCTSIB testing is limited to the head-neutral eyes-open and -closed positions. Our group has observed and reported that the eyes-closed balance position is associated with the identification of greater pathology of CoP than with the eyes open [8,12,13,14,15]. Eyes-open postural testing is associated with an increase in EEG arousal, while low EEG arousal occurs with the eyes closed [16]. The differences between eyes being open or closed are due to the cortical processing of visual inputs, which we did not want to include with our baseline measurement of balance in brain injury patients [17]. Furthermore, individuals with a concussion history demonstrate a significantly greater CoP speed dual-task cost with their eyes closed but not with eyes open [18]. We wanted to remove the vestibular and visual influences that contribute to maintaining balance in order to quantify the role of neck proprioception-related balance influences. We therefore wanted to compare the standardized head-neutral eyes-closed positions of the mCTSIB to postures involving head position differences with the eyes closed.

We wanted to make these comparisons using CDP with technology that met the minimal standards of the ISPGR.

We hypothesized that postural stability scores would be compromised with changes in head positions. Furthermore, we aimed to demonstrate that head positions off the neutral plane would provide novel biomarkers that would identify and differentiate subjects suffering from PCS from healthy normal subjects. We were successful in our goals and have identified novel biomarkers that might be used in both diagnostic and therapeutic protocols addressing concussion.

## 2. Materials and Methods

Computerized dynamic posturographic measurement of stability scores associated with induced head positional changes were collected from 575 concussion patients and 60 healthy normal subjects. This retrospective study (CI: 20200808001) was authorized by the Carrick Institute for Graduate Studies IRB (Office for Human Research Protections, Department of Health and Human Services, USA IRB00011811, IORG0009941) (Study#: 20200808001) and conducted in compliance with the principles of the Declaration of Helsinki.

The demographics and anthropometric characteristics for all the included healthy controls are detailed in Table 1.

The 575 concussion subjects were clinically diagnosed as having an mTBI by board-certified neurologists within 6 months of testing and continued to suffer from PCS. The demographics and anthropometric characteristics for these subjects are detailed in Table 2.

All subjects underwent testing by trained and qualified clinical neurologists in a single tertiary clinical setting with constantly maintained environmental conditions and testing protocols. Age, height, mass and BMI were verified to be similar between the two groups by *t*-tests with significance set at *p* < 0.05. We did not consider a gender bias as gender differences in CoP measures can be attributed to differences in anthropometry, in particular height, with no statistical differences between genders identified [19]. All subjects performed the 4 tests in bare feet, comprising the modified Clinical Test of Sensory Integration in Balance (mCTSIB) protocol plus an additional 4 head positional tests twice (a practice test followed by a recorded test). The 4 test conditions of the mCTSIB are Normal Stability (stable surface) Eyes Open (NSEO) and Eyes Closed (NSEC); and Perturbed Stability (compliant surface) Eyes Open (PSEO) and Eyes Closed (PSEC). The additional tests were performed with the same perturbed stability in the Eyes Closed Right Head Turn (PSECRT), Eyes Closed Left Head Turn (PSECLT), Eyes Closed Head Flexed (PSECHF) and Eyes Closed Head Extended (PSECHE) positions. Subjects were asked to actively rotate their heads maximally (within a comfortable personal range that did not cause discomfort) to a directed position (yaw and pitch) with their eyes open and to hold their head in this position and close their eyes for the duration of the test. 

Before performing the repetitions, each subject underwent the entire test sequences before the final recorded test. They also spent a minimum of 120 s standing on the foam cushion used in the perturbed stability tests to remove possible learning effects [15]. CoP data were acquired using strain-gaged force platform-based computerized dynamic posturography (CDP) CAPS® systems (Vestibular Technologies, LLC, Cheyenne, WY, USA), which also provided the balance measurements data used in the analysis. These systems have been shown to exceed the accuracy, precision and resolution recommended by the International Standardization Committee for Clinical Stabilometry of the International Society for Posture and Gait Research (ISPGR) [13]. We used an acquisition frequency of 64.011 Hz, 20-bit resolution with simultaneous sampling of all channels, up-sampling to 1000 Hz by interpolation via the FFT method and 5-s pre-test and 20-s test acquisition durations. We considered only the stability score (indicating the subject’s ability to maintain balance during the test, i.e., the minimum percentage of the theoretical limit of stability that the subject has left available to avoid falling at any time during the test). 

The concept behind the stability score is to consider how much sway a subject has left before falling, with the concept that if the subject sways as much as the limit of stability (LoS—the distance from the average position beyond which the subject is unable to maintain balance), then his/her stability score is 0% (there is no amount of sway left), and if the subject has no sway, then his/her stability score (SS) is 100% (100% of the LoS is left as possible sway). The formula utilized to calculate the stability score in this study is:

$$\mathrm{SS}=100*\frac{\mathrm{LoS}-\mathrm{Sway}}{\mathrm{LoS}}=100*\left(1-\frac{\mathrm{Sway}}{\mathrm{LoS}}\right)$$

We consider that the center of mass of a standing subject with arms to the side is at 0.5527 of the subject’s height, and that a person in normal stance is able to sway 6.25° degrees in every direction from the neutral [20]. Given these values, with the assumption of equal possible sway in any direction from the center, the theoretical LoS (distance from the center) is:

Theoretical LoS=0.55∗h∗sin(6.25°)

We identified the largest sway considering all directions. Sway is the movement of the center of mass (CoM), and its measurement requires measuring all of the inertial properties and positions of all body parts. Posturography testing uses the CoP instead of the CoM, assuming they coincide, which is true when the body is at rest. We also used the largest 95% confidence sway in any direction (1.96 the maximum standard deviation of the CoP coordinates). This can be calculated using the variance–covariance matrix of the CoP coordinates.

$$C_{\mathrm{xy}}=\left[\begin{array}{cc} \sigma_{x}^{2} & \sigma_{\mathrm{xy}}\\ \sigma_{\mathrm{xy}} & \sigma_{y}^{2} \end{array}\right]=\frac{1}{\left(n-1\right)}\left[\begin{array}{cc} S_{x^{2}} & S_{\mathrm{xy}}\\ S_{\mathrm{xy}} & S_{y^{2}} \end{array}\right]$$

where

$$\bar{X}=\frac{\sum X_{i}}{n}$$

$$\bar{Y}=\frac{\sum Y_{i}}{n}$$

$$S_{x^{2}}=\sum {\left(X_{i}-\bar{X}\right)}^{2}$$

$$S_{y^{2}}=\sum {\left(Y_{i}-\bar{Y}\right)}^{2}$$

$$S_{\mathrm{xy}}=\sum \left(X_{i}-\bar{X}\right)\left(Y_{i}-\bar{Y}\right)$$

and $\left(X_{i},Y_{i}\right)\;$are the x and y coordinates of each CoP sampled position, respectively, and *n* is the overall number of sampled CoP positions. The covariance matrix is symmetric positive definite, and furthermore, it is a tensor. Therefore, its eigenvalues are all real and positive and the eigenvectors that belong to the distinct eigenvalues are orthogonal. Therefore, it is always possible to diagonalize it, i.e., to find a rotation of the coordinates that will produce a diagonal matrix.

The eigenvalues of the covariance matrix are the maximum and minimum (principal components) of the variance. Their square roots are the overall maximum and minimum standard deviations of the data if one considers the CoP data in any 2D orthogonal coordinate system. The eigenvectors represent the directions along which the maximum and minimum variance and standard deviations occur. We calculated the largest 95% confidence sway in any direction by taking 1.96 times the square root of the largest of the 2 eigenvalues of the covariance matrix. The 95% confidence interval is centered around the mean and extends by 1.96 below and over the mean, so the largest 95% confidence back and forth sway around the average CoP/CoM position is 2 × 1.96-times the square root of the largest of the 2 eigenvalues of the covariance matrix.

With this information, we computed the stability score:

$$\mathrm{SS}=100*\frac{\mathrm{LoS}-\mathrm{Sway}}{\mathrm{LoS}}=100*\left(1-\frac{\mathrm{Sway}}{\mathrm{LoS}}\right)=100*\left(1-\frac{\mathrm{Largest}95\%\mathrm{confidenceSway}}{\mathrm{TheoreticalLoS}}\right)$$

Note that because the theoretical LoS is calculated using the height of the subject, although the sway of the subject is related to its height (a taller subject sways more in terms of distance than a shorter subject, although the sway in terms of angles from the vertical will be the same), the SS does not depend on the height (it is a measure normalized by the subject’s height, and it is an adimensional quantity). The stability score has several advantages as a measure: it considers the maximum sway (highest postural instability) in any direction, not just antero-posterior or medio-lateral; it normalizes, albeit indirectly, the sway by the subject’s height, eliminating a major source of difference between subjects caused by different anthropometry, and has been shown to have excellent reliability and validity [21].

The statistical analysis was done in STATA 16.1 (StataCorp, College Station, TX, USA) and consisted of general linear models, linear and logistic regression and multiple *t*-tests, all with alpha < 0.05 and a power maintained at 80%.

## 3. Results

Table 3 describes the stability score (mean and SEM) for the different head positions for the two groups (healthy controls and patients).

As expected, the mCTSIB demonstrated statistically significant differences between the eyes-open and -closed normal and perturbed surfaces with the head neutral in both healthy controls and concussion patients. This testing added nothing new to our investigation and reinforced our choice to test head rotations in the eyes-closed perturbed position as previously discussed (Table 4, Table 5, Table 6 and Table 7).

A general linear model within subjects comparing eyes-closed head rotation stability scores to the eyes-closed neutral head position stability scores on perturbed surfaces revealed statistically significant differences between the head rotations in right and left yaw in the concussion patients but not in the normal subjects. Both concussion patients and normal healthy controls had statistically significant differences between the head neutral and head extended positions stability scores. Normal healthy controls demonstrated statistically significant differences between head flexion and head neutral positions stability scores that were not seen in the concussion patients (Table 8, Table 9, Table 10 and Table 11).

The differences between the head neutral and right head turned postures were statistically significant in concussion subjects using a paired *t*-test (*t* (574) = 2.0259, *p* < 0.043), demonstrating less stability with the head turned. A linear regression model predicting the stability score of a concussion subject with a right head turn by the head neutral position was statistically significant, with less stability associated with the head turn (F(1, 573) = 667.53, [95% CI 0.6976099–0.8124015], *p* ≤ 0.001). We expect an increase of 0.76 units in the stability score of a right head turn for every increase of 1 unit of stability in the head neutral position. There are extremely strong substantive significant effect sizes (R^2^ = 0.5381, η^2^ = 0.538).

We desired to have the results of all of our linear regression models made more tangible by computing predicted or expected values for hypothetical or prototypical cases [22]. We calculated adjusted predictions from the predictions of our previously fit model at hypothetical fixed values of some covariates and averaging or otherwise integrating over the remaining covariates. We specified the values for each of the hypothetical independent variables in the model at 0–100, at intervals of 20, and then computed the probability of the event occurring for an individual who has those values. We then measured the effect on the conditional mean of y of a change in each of the hypothetical regressors, with the effect being equal to the relevant slope coefficient. We plotted these results as adjusted predictions that should not be confused with plots of the linear regression model.

**Figure 1:**
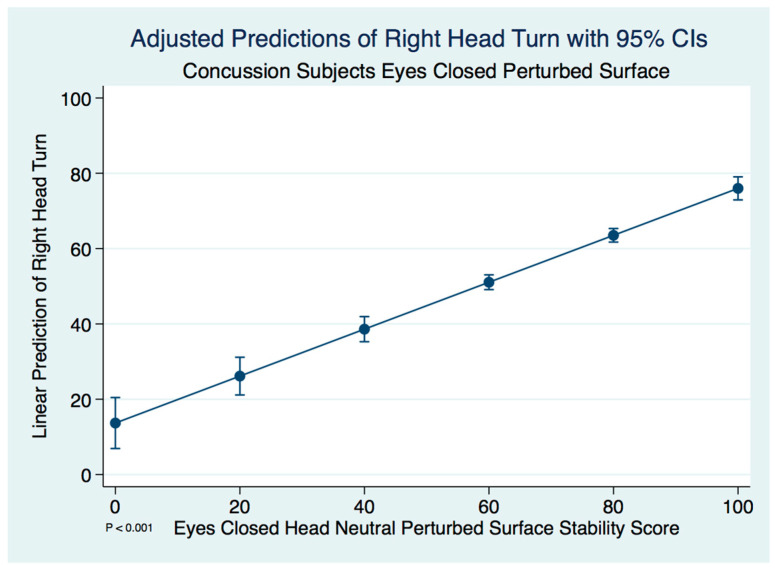
Adjusted predictions of right head turn stability scores in concussion subjects.

The adjusted predictions of right head turn stability scores with 95% CIs are demonstrated in Figure 1.

However, there were no significant differences between the head neutral and right head turned postures in healthy control subjects using a paired *t*-test (*t* (59) = −1.0339, *p* = 0.305).

**Figure 2:**
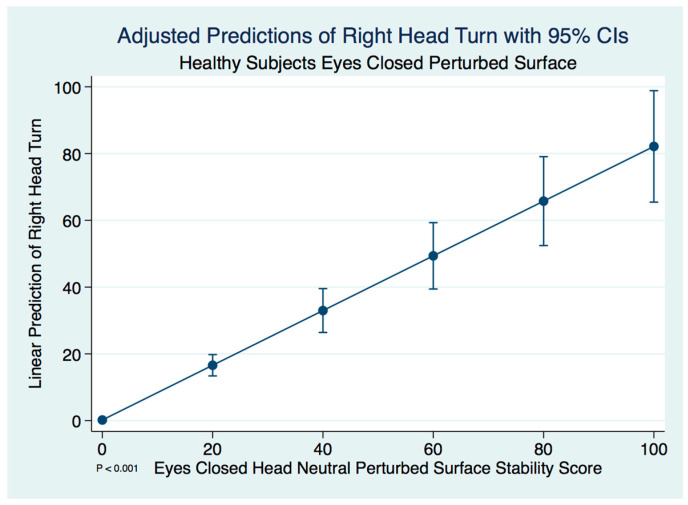
Adjusted predictions of right head turn stability scores in healthy normal controls.

A linear regression model predicting the stability score of a healthy control subject with a right head turn by the head neutral position was statistically significant (F(1, 58) = 82.68, [95% CI 0.6372409–0.9970173], *p* ≤ 0.001). We expect an increase of 0.82 units in the stability score of a right head turn for every increase of 1 unit of stability in the head neutral position. There are extremely strong substantive significant effect sizes (R^2^ = 0.587, η^2^ = 0.588). The adjusted predictions of right head turn stability scores with 95% CIs are demonstrated in Figure 2.

**Figure 3:**
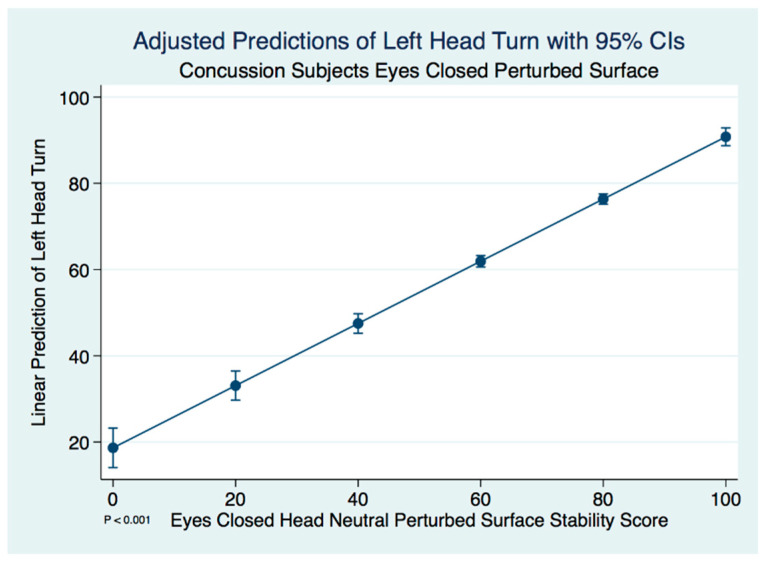
Adjusted predictions of left head turn stability scores in concussion subjects.

Again, we found significant differences in the concussion subjects with a left head turn compared to the head neutral position on a perturbed surface with the eyes closed, this time using a paired *t*-test (*t* (574) = 2.2536, *p* = 0.025). A linear regression model predicting the stability score of a concussion subject with a left head turn by the head neutral position was statistically significant (F(1, 573) = 525.19, [95% CI 0.6594581–0.7830927], *p* ≤ 0.001). We expect an increase of 0.72 units in the stability score of a left head turn for every increase of 1 unit of stability in the head neutral position. There are extremely strong substantive significant effect sizes (R^2^ = 0.478, η^2^ = 0.478. The adjusted predictions of left head turn stability scores for the concussion subjects with 95% CIs are demonstrated in Figure 3.

**Figure 4:**
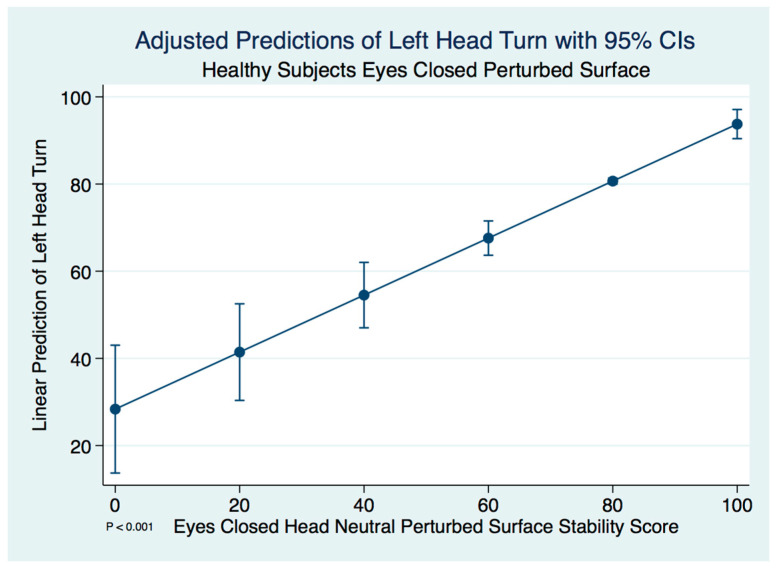
Adjusted predictions of left head turn stability scores in healthy normal controls.

Unlike the concussion subjects, we did not find any significant differences between the head neutral and left head turned postures in healthy control subjects using a paired *t*-test (*t* (59) = −0.2624, *p* = 0.794). A linear regression model predicting the stability score of a healthy control subject with a left head turn by the head neutral position was statistically significant (F(1, 58) = 53.23, [95% CI 0.4745143–0.8333282], *p* ≤ 0.001). We expect an increase of 0.65 units in the stability score of a left head turn for every increase of 1 unit of stability in the head neutral position. There are extremely strong substantive significant effect sizes (R^2^ = 0.479, η^2^ = 0.479). The adjusted predictions of left head turn stability scores with 95% CIs are demonstrated in Figure 4.

**Figure 5:**
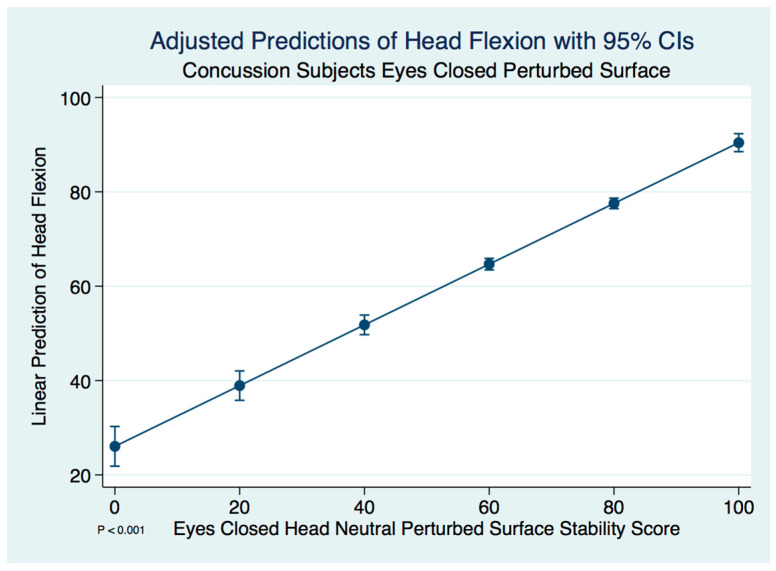
Adjusted predictions of head flexion stability scores in concussion subjects.

We did not find a significant difference in the stability scores in the concussion subjects when the head was flexed forward compared to the head neutral position on a perturbed surface with the eyes closed using a paired *t*-test (*t* (574) = −0.8276, *p* = 0.4082). A linear regression model predicting the stability score of a concussion subject with head flexion by the head neutral position was statistically significant (F(1, 573) = 492.29, [95% CI 0.5865769–0.7005139], *p* ≤ 0.001). We expect an increase of 0.64 units in the stability score with head flexion for every increase of 1 unit of stability in the head neutral position. There are extremely strong substantive significant effect sizes (R^2^ = 0.462, η^2^ = 0.462). The adjusted predictions of head flexion stability scores for the concussion subjects with 95% CIs are demonstrated in Figure 5.

**Figure 6:**
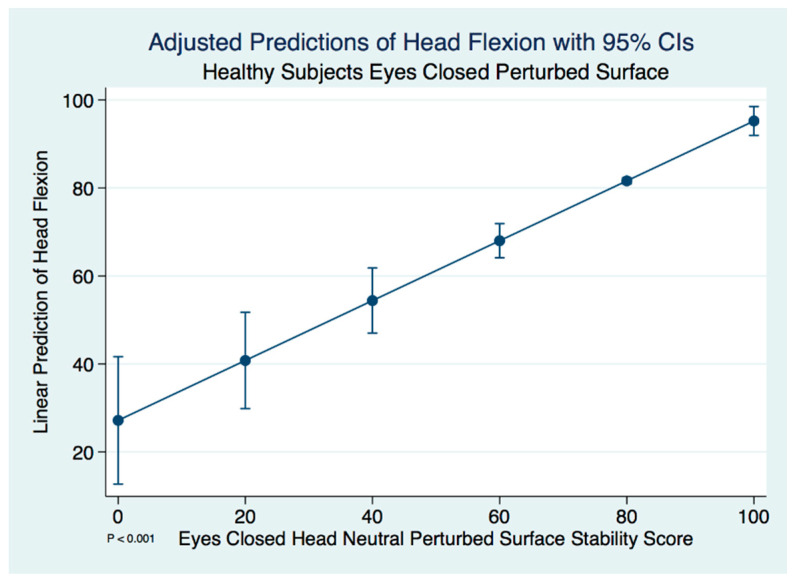
Adjusted predictions of head flexion stability scores in healthy normal controls.

Unlike the concussion subjects, we did find significant differences between the head neutral and head flexed postures in healthy control subjects using a paired *t*-test (*t* (59) = −3.247, *p* = 0.002). A linear regression model predicting the stability score of a healthy control subject with head flexed by the head neutral position was statistically significant (F(1, 58) = 59.23, [95% CI 0.5035146–0.8574998], *p* ≤ 0.001). We expect an increase of 0.68 units in the stability score of head flexed for every increase of 1 unit of stability in the head neutral position. There are extremely strong substantive significant effect sizes (R^2^ = 0.505, η^2^ = 0.505). The adjusted predictions of head flexion stability scores in healthy controls with 95% CIs are demonstrated in Figure 6.

**Figure 7:**
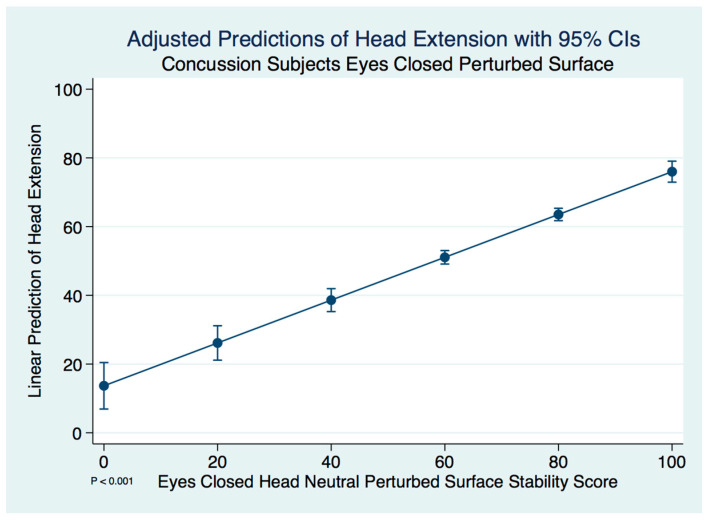
Adjusted predictions of head extension stability scores in concussion subjects.

By far the greatest differences in posture with head movement were seen with extension of the head. We found a significant difference in the stability scores in the concussion subjects when the head was extended compared to the head neutral position on a perturbed surface with the eyes closed using a paired *t*-test (*t* (574) = 15.1967, *p* = 0.0000). A linear regression model predicting the stability score of a concussion subject with head extension by the head neutral position was statistically significant (F(1, 573) = 178.78, [95% CI 0.5315742–0.7146363], *p* ≤ 0.001). We expect an increase of 0.62 units in the stability score with head extension for every increase of 1 unit of stability in the head neutral position. There are extremely strong substantive significant effect sizes (R^2^ = 0.238, η^2^ = 0.238). The adjusted predictions of head extension stability scores for the concussion subjects with 95% CIs are demonstrated in Figure 7.

**Figure 8:**
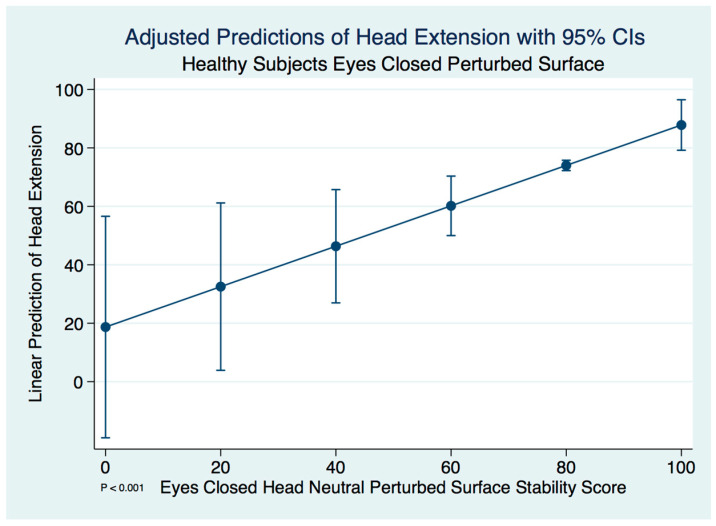
Adjusted predictions of head extension stability scores in healthy normal controls.

Similar to our findings of head extension in concussion subjects, we did find significant differences between the head neutral and head extended postures in healthy control subjects using a paired *t*-test (*t* (59) = 8.1435, *p* = 0.0000). A linear regression model predicting the stability score of a healthy control subject extending the neck by the head neutral position was statistically significant (F(1, 58) = 8.91, [95% CI 0.2277891–1.155032], *p* = 0.004). We expect an increase of 0.69 units in the stability score of head extended for every increase of 1 unit of stability in the head neutral position. There are extremely strong substantive significant effect sizes (R^2^ = 0.133, η^2^ = 0.133). The adjusted predictions of head extension stability scores in healthy controls with 95% CIs are demonstrated in Figure 8.

**Figure 9:**
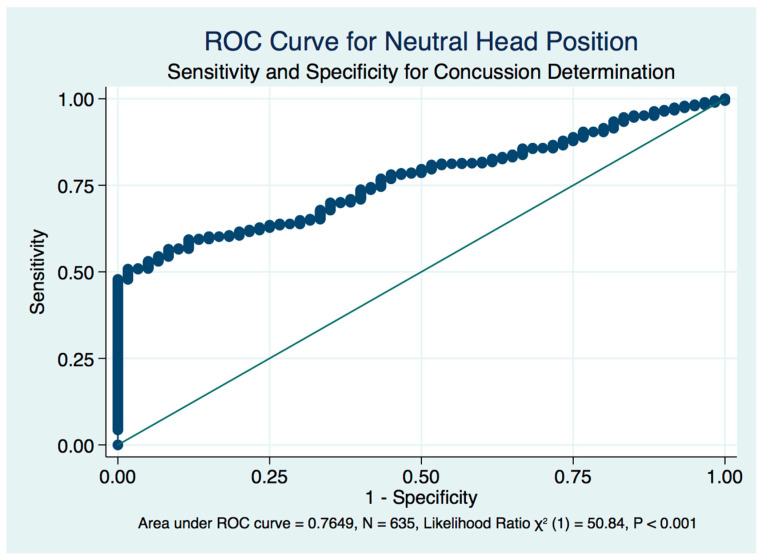
Receiver operating characteristic (ROC) curve for head neutral, eyes closed perturbed testing in concussion determination.

**Figure 10:**
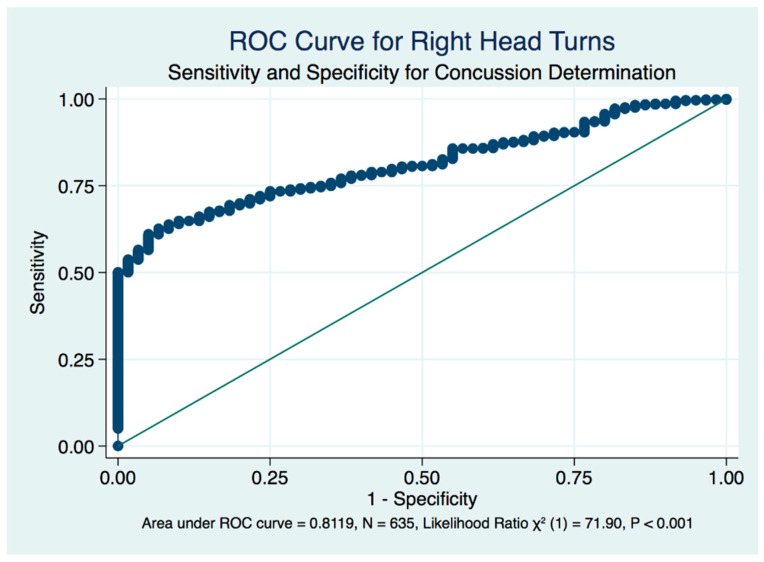
ROC curve for right head turn, eyes closed perturbed testing in concussion determination.

**Figure 11:**
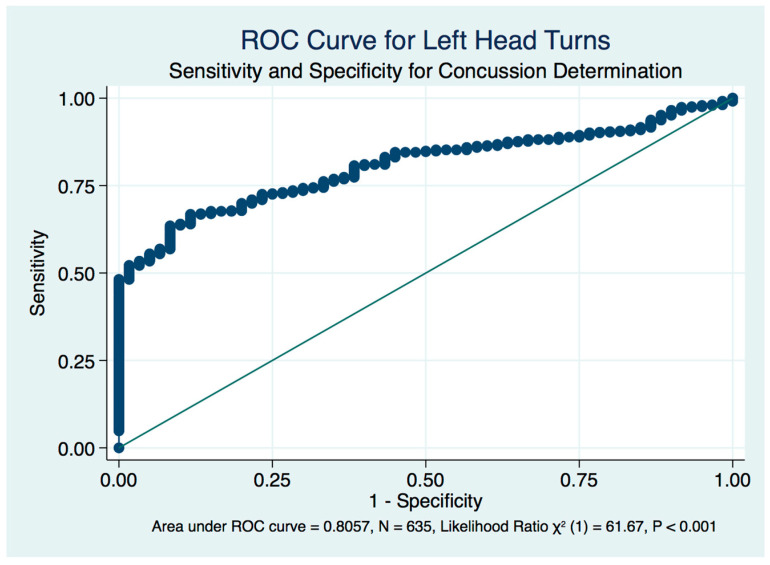
ROC curve for left head turn, eyes closed perturbed testing in concussion determination.

**Figure 12:**
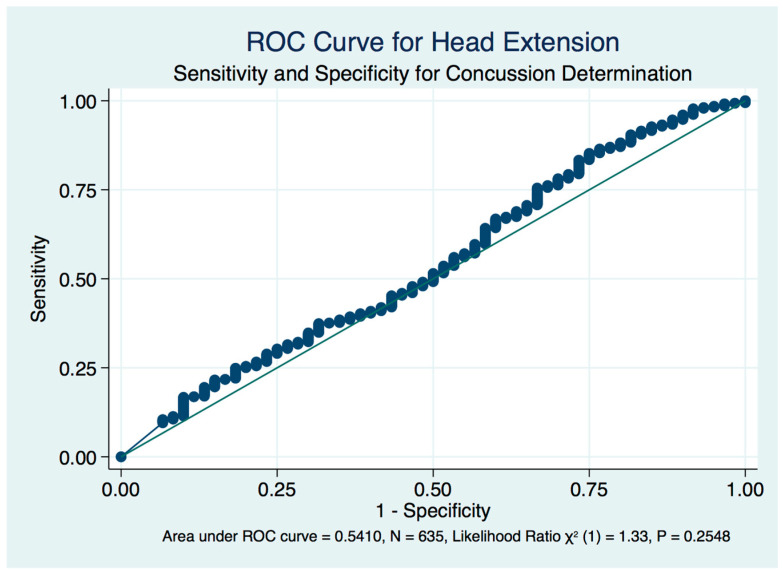
ROC curve for head extension, eyes closed perturbed testing in concussion determination.

We plotted receiver operating characteristic curves (ROC) of the true positively identified concussion cases against the false positive rate of concussion identification by the stability scores induced by head position. The standardized neutral head position in the mCTSIB provides only a fair but significant diagnostic biomarker of concussion (area under the curve (AUC) = 0.7649, LR χ^2^ (1) = 50.84, *p* < 0.001) (Figure 9), whereas both right (AUC = 0.8119, LR χ^2^ (1) = 71.90, *p* < 0.001) (Figure 10) and left (AUC = 0.8057, LR χ^2^ (1) = 61.67, *p* < 0.001) (Figure 11) head turns provided good accuracy and statistically significant diagnostic biomarkers for concussion. Although head extension decreases the stability score of both healthy controls and concussion subjects with statistical significance compared to the neutral head position, it is non-significant and fails as a diagnostic test that might be used as a biomarker to identify a concussion (Figure 12). 

## 4. Discussion

Balance loss or compromise may be caused by neurological disorders that increase the time-delay in the neuromuscular system [23]. We have demonstrated that the position of the head and neck induced by statically maintained head turns is associated with significantly lower stability scores than the standardized head neutral position of the mCTSIB in PCS subjects but not in normal healthy controls. This phenomenon may serve as a diagnostic biomarker to differentiate PCS subjects from normal one as well as serving as a measurement with which to quantify function or the success or failure of a treatment.

Sport-related concussion (SRC) is associated with inconsistency in clinical assessment integrity, largely focusing on function of neurocognition, symptom scores and postural stability [24]. The observed biomarkers of increased postural instability with head turns in PCS can serve to decrease inconsistency by establishing a physiological biomarker that is not associated with subjective variability for a myriad of reasons [24].

Concussion represents a functional rather than a structural injury that results in shear stress to the brain and neck [25]. The standardized mCTSIB head neutral postural examinations are not adequate to identify individuals that have suffered a concussion. However, this study has identified significant differences in the postural stability scores with head turns in PCS subjects that differentiates them from normal healthy controls. We suggest that CDP examinations include an extended version of the mCTSIB that will include head turns. The involvement of head and neck influences on postural stability would have been missed in our large sample of subjects if we did not include the additional postural testing positions associated with head turns. This has significant clinical applications that might address the proprioceptive system in concussion rehabilitation with biomarkers that can measure the success or failure of a treatment.

There has been a significant amount of attention given to the impairment of proprioception integration after a concussion [26,27,28,29,30,31,32,33,34,35]. There have been no good biomarkers that might identify the consequences of neck and head integration of function after a concussion. We feel that the utilization of the measurement of postural stability that includes head positional changes as we have described will improve the diagnostic and therapeutic clarity needed in a multimodal approach to the complexity of understanding of mTBI. We recommend the use of an extended mCTSIB rather than the standardized and limited mCTSIB in the postural evaluation of concussion patients. 

Head positions are common postures recognized by clinicians, yet their functional contribution to balance has not been included in the standardized mCTSIB [36]. This study has identified the significant differences between balance testing in the head neutral position and with a variety of head positions. There are no significant differences between head and shoulder postures between genders [37] and our findings suggest that there are also no functional changes or gender bias in balance performance associated with changes in head position. Proprioception is changed by head position, as is the pattern of breathing and muscle activity of the human body [38]. This investigation has demonstrated the importance of including different head positions in the evaluation of balance, especially after a head injury. Lower stability scores after traumatic brain injury and stroke are consistent with abnormal supersegmental integration of proprioception. Identification of proprioceptive-based sensorimotor pathology of stability scores induced by head positional changes can lead to appropriate therapeutic applications that might be quantified by changes in the stability scores after treatment. For example, the functional level of brain function after a stroke of any kind can be improved by proprioceptive neuromuscular facilitation [39]. 

Orthostatic posture is influenced by head stabilization under proprioceptive control that is changed with altered neck proprioception associated with positional changes [40]. We have identified the sensitivity and specificity of stability scores with changes in head position in concussion patients. Changes in muscle tone in the neck result in movement angular errors due to multisensory integrational changes affecting a full-body geometrical representation necessary to plan movements and balance [41]. There is a significant impairment of balance between patients that have whiplash-associated disorders affecting neck proprioception and healthy controls [42]. Our findings promote the inclusion of head positional changes in balance testing to identify neck proprioceptive differences that are not identified in the head neutral position.

Sensorimotor control disturbances are associated with altered cervical proprioception and disturbances of postural stability in those with neck disorders [43]. Even the sensorimotor development of the brain in neonates is dependent upon movement that is ultimately dependent upon joint position [44]. Joint positional changes due to head position may uncover deficits in postural stability scores due to brain pathology of function. For instance, children with cerebral palsy have a deficit in postural control and head stability compared to normal healthy children, thus further emphasizing the need to challenge stability with head positional changes during balance testing [45]. The use of the standardized mCTSIB has continued the 19th century observations of proprioceptive integration described by Moritz Romberg without much change [46]. Our findings of significant differences in postural stability scores with head turns strongly suggests an extension of the mCTSIB and upgrade of testing that has been consistent since the 1800s.

## 5. Conclusions

The standardized mCTSIB that is limited to a head neutral position may not recognize decreases in postural stability scores that are associated with head positional change. Patients that have suffered a concussion have associated decreased postural stability scores with eyes-closed changes of head positions. Head positional postures can be considered to be biomarkers to differentiate the postural stability scores of concussion patients from those of healthy normal subjects with statistical and substantive significance. We recommend the use of head positional testing by creating an extended mCTSIB, especially in the evaluation of individuals that have suffered a brain injury. 

## Figures and Tables

**Table 1:** Demographics and anthropometric characteristics for all the included healthy controls (number of subjects, mean ± SEM and range). Values are grouped by sex and the breakdown between the three age groups is also included.

	Age (Years)	Height (m)	Mass (kg)	BMI (kg/m^2^)
Males (35)	33.06 ± 1.80 20–64	1.79 ± 0.01 1.62–2.00	87.33 ± 2.61 65.00–149.30	27.34 ± 0.66 21.69–41.14
Females (25)	29.84 ± 1.86 19–52	1.65 ± 0.01 1.52–1.80	70.15 ± 2.65 51.54–101.39	25.84 ± 1.01 18.89–40.61
18–25 (20)	23.55 ± 0.41 19–25	1.72 ± 0.02 1.55–1.94	75.81 ± 3.47 53.97–101.39	25.70 ± 1.10 19.92–40.61
26–34 (24)	29.46 ± 0.62 26–34	1.74 ± 0.02 1.52–2.00	85.04 ± 3.99 57.24–149.30	27.73 ± 0.92 20.37–41.14
35–65 (16)	45.31 ± 2.41 35–64	1.72 ± 0.02 1.57–1.86	78.27 ± 3.08 53.96–104.01	26.45 ± 0.86 18.89–31.10
All (60)	31.72 ± 1.31 19–64	1.73 ± 0.01 1.52–2.00	80.16 ± 2.17 53.96–149.30	26.71 ± 0.57 18.89–41.14

Stability score repeated measure GLM by sex and age group. No effect of sex: *p* = 0.094, partial Eta squared = 0.049, power = 0.387. No effect of age group: *p* = 0.164, partial Eta squared = 0.062, power = 0.372.

**Table 2:** Demographics and anthropometric characteristics for the patients (number of subjects, mean ± SEM and range). Values are grouped by sex and the breakdown between the three age groups are also included.

	Age (Years)	Height (m)	Mass (kg)	BMI (kg/m^2^)
Males (335)	33.88 ± 0.67 18–65	1.82 ± 0.01 1.52–2.23	86.60 ± 0.83 52.02–173.66	26.17 ± 0.214 15.38–47.84
Females (240)	37.61 ± 0.87 18–65	1.66 ± 0.01 1.48–1.88	68.13 ± 0.93 40.03–122.90	24.77 ± 0.32 15.15–42.94
18–25 (175)	22.31 ± 0.16 18–25	1.79 ± 0.01 1.52–2.23	78.38 ± 1.40 48.62–173.66	24.40 ± 0.29 16.79–42.94
26–34 (152)	29.72 ± 0.21 26–34	1.76 ± 0.01 1.50–2.12	78.77 ± 1.41 46.11–124.47	25.22 ± 0.35 17.20–39.37
35–65 (248)	48.21 ± 0.55 35–65	1.72 ± 6.84 1.48–2.06	79.33 ± 1.06 40.03–131.77	26.65 ± 0.30 15.15–47.84
All (575)	35.44 ± 0.54 18–65	1.75 ± 0.01 1.48–2.23	78.89 ± 0.73 40.03–173.66	25.59 ± 0.19 15.15–47.84

Stability score repeated measure GLM by sex and age group. Effect of sex: *p* = 0.041, partial Eta squared = 0.007, power = 0.536. Effect of age group: *p* = 0.009, partial Eta squared = 0.016, power = 0.788.

**Table 3:** Stability score (mean and SEM) for the different head positions for the two groups (healthy controls and patients). The repeated measures GLM results of the differences from the head neutral reference position for each group are also included.

	Head Position	Mean	Std Error	Significance *p*	Partial Eta Squared	Observed Power
Controls (*n* = 60)	Head neutral (reference)	81.679	0.447			
	Head Right	82.007	0.476	0.305	0.019	0.174
	Head Left	81.769	0.422	0.794	0.001	0.058
	Head Flexed	82.761	0.428	0.002	0.152	0.891
	Head Extended	75.164	0.847	0.000	0.529	1.000
Patients (*n* = 575)	Head neutral (reference)	71.753	0.747			
	Head Right	70.632	0.769	0.043	0.007	0.525
	Head Left	70.401	0.779	0.025	0.009	0.614
	Head Flexed	72.236	0.707	0.408	0.001	0.131
	Head Extended	58.391	0.954	0.000	0.287	1.000

**Table 4:** Healthy controls’ postural stability scores head neutral mCTSIB.

Test	Mean	Std. Error	95% Confidence Interval	
			Lower Bound	Upper Bound
NSEO-HN	94.550	0.227	94.096	95.003
NSEC-HN	92.651	0.268	92.115	93.187
PSEO-HN	89.968	0.267	89.433	90.503
PSEC-HN	81.679	0.447	80.785	82.574

**Table 5:** Tests of within-Subjects Contrasts for Healthy Controls’ Postural Stability Scores Head Neutral mCTSIB.

Source	Test	Type III Sum of Squares	df	Mean Square	F	Sig.	Partial Eta Squared	Noncent. Parameter	Observed Power
Test	NSEO-HN vs. NSEC-HN	216.376	1	216.376	49.873	0.000	0.458	49.873	1.000
	NSEC-HN vs. PSEO-HN	431.840	1	431.840	55.045	0.000	0.483	55.045	1.000
	PSEO-HN vs. PSEC-HN	4121.937	1	4121.937	325.927	0.000	0.847	325.927	1.000
Error (Test)	NSEO-HN vs. NSEC-HN	255.974	59	4.339					
	NSEC-HN vs. PSEO-HN	462.863	59	7.845					
	PSEO-HN vs. PSEC-HN	746.161	59	12.647					

Computed using alpha = 0.05. Normal Surface Eyes Open (NSEO); Normal Surface Eyes Closed (NSEC); Perturbed Surface Eyes Open (PSEO); Perturbed Surface Eyes Closed (PSEC); Head Neutral (HN).

**Table 6:** Concussion patients’ postural stability scores head neutral mCTSIB.

Test	Mean	Std. Error	95% Confidence Interval	
			Lower Bound	Upper Bound
NSEO-HN	90.412	0.359	89.708	91.117
NSEC-HN	89.075	0.476	88.140	90.009
PSEO-HN	82.693	0.474	81.761	83.624
PSEC-HN	71.753	0.747	70.286	73.220

**Table 7:** Tests of within-Subjects Contrasts for Concussion Patients’ Postural Stability Scores Head Neutral mCTSIB.

Source	Test	Type III Sum of Squares	df	Mean Square	F	Sig.	Partial Eta Squared	Noncent. Parameter	Observed Power ^a^
Test	NSEO-HN vs. NSEC-HN	1028.855	1	1028.855	19.339	0.000	0.033	19.339	0.992
	NSEC-HN vs. PSEO-HN	23,418.979	1	23,418.979	249.292	0.000	0.303	249.292	1.000
	PSEO-HN vs. PSEC-HN	68,812.928	1	68,812.928	310.301	0.000	0.351	310.301	1.000
Error (Test)	NSEO-HN vs. NSEC-HN	30,537.599	574	53.201					
	NSEC-HN vs. PSEO-HN	53,922.698	574	93.942					
	PSEO-HN vs. PSEC-HN	127,291.478	574	221.762					

^a^ Computed using alpha = 0.05. Normal Surface Eyes Open (NSEO); Normal Surface Eyes Closed (NSEC); Perturbed Surface Eyes Open (PSEO); Perturbed Surface Eyes Closed (PSEC); Head Neutral (HN).

**Table 8:** Healthy controls’ postural stability scores with head rotation.

Test	Mean	Std. Error	95% Confidence Interval	
			Lower Bound	Upper Bound
PSEC-HN	81.679	0.447	80.785	82.574
PSEC-HR	82.007	0.476	81.054	82.960
PSEC-HL	81.769	0.422	80.924	82.614
PSEC-HF	82.761	0.428	81.905	83.617
PSEC-HE	75.164	0.847	73.470	76.858

**Table 9:** Tests of within-Subjects Contrasts for Healthy Controls’ Postural Stability Scores with Head Rotation.

Source	Test	Type III Sum of Squares	df	Mean Square	F	Sig.	Partial Eta Squared	Noncent. Parameter	Observed Power ^a^
Test	PSEC-HR vs. PSEC-HN	6.426	1	6.426	1.069	0.305	0.018	1.069	0.174
	PSEC-HL vs. PSEC-HN	0.483	1	0.483	0.069	0.794	0.001	0.069	0.058
	PSEC-HF vs. PSEC-HN	70.179	1	70.179	10.546	0.002	0.152	10.546	0.891
	PSEC-HE vs. PSEC-HN	2547.391	1	2547.391	66.317	0.000	0.529	66.317	1.000
Error (Test)	PSEC-HR vs. PSEC-HN	354.697	59	6.012					
	PSEC-HL vs. PSEC-HN	413.943	59	7.016					
	PSEC-HF vs. PSEC-HN	392.633	59	6.655					
	PSEC-HE vs. PSEC-HN	2266.327	59	38.412					

^a^ Computed using alpha = 0.05. Perturbed Surface Eyes Open (PSEC); Head Right (HR); Head Left (HL); Head Flexed (HF); Head Extended (HE).

**Table 10:** Concussion patients’ postural stability scores with head rotation.

Measure: SS				
Test	Mean	Std. Error	95% Confidence Interval	
			Lower Bound	Upper Bound
PSEC-HN	71.753	0.747	70.286	73.220
PSEC-HR	70.632	0.769	69.122	72.142
PSEC-HL	70.401	0.779	68.872	71.931
PSEC-HF	72.236	0.707	70.847	73.624
PSEC-HE	58.391	0.954	56.516	60.265

**Table 11:** Tests of within-Subjects Contrasts for Concussion Patients’ Postural Stability Scores with Head Rotation.

Source	Test	Type III Sum of Squares	df	Mean Square	F	Sig.	Partial Eta Squared	Noncent. Parameter	Observed Power ^a^
Test	PSEC-HR vs. PSEC-HN	722.992	1	722.992	4.104	0.043	0.007	4.104	0.525
	PSEC-HL vs. PSEC-HN	1050.861	1	1050.861	5.079	0.025	0.009	5.079	0.614
	PSEC-HF vs. PSEC-HN	133.792	1	133.792	0.685	0.408	0.001	0.685	0.131
	PSEC-HE vs. PSEC-HN	102,672.138	1	102,672.138	230.938	0.000	0.287	230.938	1.000
Error (Test)	PSEC-HR vs. PSEC-HN	101,111.042	574	176.152					
	PSEC-HL vs. PSEC-HN	118,773.031	574	206.922					
	PSEC-HF vs. PSEC-HN	112,114.141	574	195.321					
	PSEC-HE vs. PSEC-HN	255,192.758	574	444.587					

^a^ Computed using alpha = 0.05. Perturbed Surface Eyes Open (PSEC); Head Right (HR); Head Left (HL); Head Flexed (HF); Head Extended (HE).
